# A Comparative Study of Newly Developed HPLC-DAD and UHPLC-UV Assays for the Determination of Posaconazole in Bulk Powder and Suspension Dosage Form

**DOI:** 10.1155/2014/241035

**Published:** 2014-09-03

**Authors:** Dalia A. Hamdy, Tarek S. Belal

**Affiliations:** Pharmaceutical Analytical Chemistry Department, Faculty of Pharmacy, Alexandria University, 1 Khartoum Square, Azarita, Alexandria 21521, Egypt

## Abstract

*Objective*. To develop and compare HPLC-DAD and UHPLC-UV assays for the quantitation of posaconazole in bulk powder and suspension dosage form. *Methods*. Posaconazole linearity range was 5–50 *μ*g/mL for both assays. For HPLC-DAD assay, samples were injected through Zorbax SB-C18 (4.6 × 250 mm, 5 *μ*m) column. The gradient elution composed of the mobile phase acetonitrile: 15 mM potassium dihydrogen orthophosphate (30 : 70 to 80 : 20, linear over 7 minutes) pumped at 1.5 mL/min. For UHPLC-UV assay, samples were injected through Kinetex-C18 (2.1 × 50 mm, 1.3 *μ*m) column. The mobile phase composed of acetonitrile: 15 mM potassium dihydrogen orthophosphate (45 : 55) pumped isocratically at 0.4 mL/min. Detection wavelength was 262 nm in both methods. *Results*. The run time was 11 and 3 minutes for HPLC-DAD and UHPLC-UV assays, respectively. Both assays were linear (*r* ^2^ > 0.999) with CV% and % error of the mean <3%. Limits of detection and quantitation were 0.82 and 2.73 *μ*g/mL for HPLC-DAD and 1.04 and 3.16 *μ*g/mL for UHPLC-UV, respectively. The methods quantitated PSZ in suspension dosage form with no observable interferences. *Conclusions*. Both assays were proven sensitive and selective according to ICH guidelines. UHPLC-UV assay exhibited some economic and chromatographic separation superiority.

## 1. Introduction

**Figure 1:**
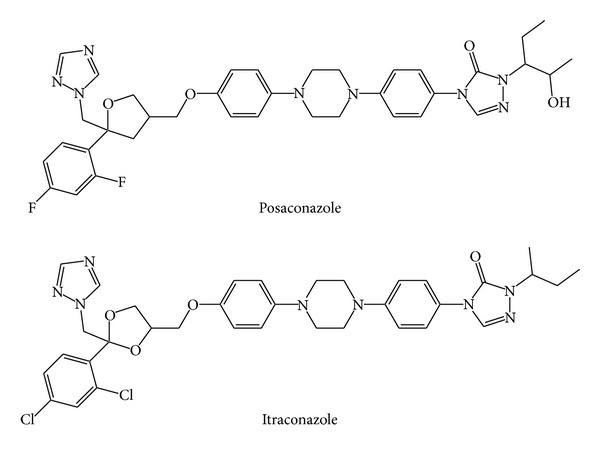
Chemical structures of posaconazole (PSZ) and the internal standard (itraconazole).

Over the last two decades, immunosuppression associated with human immunodeficiency virus, organ transplantation, and anticancer chemotherapy has resulted in a dramatic increase in the incidence of serious mucosal and systemic fungal infections [1]. Posaconazole (PSZ) (Figure 1), chemically known as 4-{p-[4-(p-{[(3*R*,5*R*)-5-(2,4-difluorophenyl)tetrahydro-5-(1*H*-1,2,4-triazol-1-ylmethyl)-3-furyl]methoxy}phenyl)-1-piperazinyl]phenyl}-1-[(1*S*,2*S*)-1-ethyl-2-hydroxypropyl]-Δ^2^-1,2,4-triazolin-5-one, is a structural analogue of itraconazole. It is commercially available in the form of oral suspension (40 mg/mL) and delayed release (100 mg) tablets. Both dosage forms can be used in the prophylactic treatment of invasive Aspergillus and Candida infections in severely immunocompromised patients thirteen years old and older [2]. Those immunocompromised patients include those with hematopoietic stem cell transplantation with graft-versus-host disease or hematologic malignancies with prolonged neutropenia from chemotherapy [3]. However, only the oral suspension has been recently indicated for the treatment of oropharyngeal candidiasis refractory to itraconazole and/or fluconazole.

Despite that PSZ oral suspension is commercially available since 2005 and delayed release 100 mg tablets since November 2013, it is not yet an official drug in any of the famous pharmacopoeias, including the British Pharmacopoeia, European Pharmacopoeia, and United States Pharmacopoeia. Nevertheless, several methods can be found in the scientific literature for its analysis in biological fluids. Most of these methods are based on liquid chromatography using different types and detection modes such as HPLC with UV detection [4–6], HPLC with fluorescence detection [7, 8], HPLC-tandem mass spectrometry (LC-MS/MS) [9–12], UPLC-UV detection [13], and UPLC-MS/MS [14]. Recently, HPLC with DAD detection was applied for the stability indicating determination of PSZ in bulk form [15]. Moreover, other separation techniques such as capillary electrophoresis [16] and micellar electrokinetic chromatography (MEKC) [17] were utilized for the quantification of PSZ in human plasma samples.

Ultra High Performance Liquid Chromatography (UHPLC) is a relatively new technique which has been introduced in 2004. UHPLC is a derivative of HPLC showing a dramatic enhancement in speed, resolution, and the sensitivity of analysis due to the use of column particle size less than 2 *μ*m. This advantageous system can operate at higher pressure with the mobile phase running at greater linear velocities as compared to HPLC. It is considered a new focal point in field of liquid chromatographic studies with significant reduction in analytical time, sample volume, and solvent consumption along with superior chromatographic separation [18, 19]. Thus it has been adopted by pharmaceutical industry for routine quality control purposes.

The fact that up till now the analysis of this drug formulation has not been tackled yet encouraged us to develop and optimize two simple, advanced, and reliable HPLC-DAD and UHPLC-UV procedures for quality control purposes. The structurally related itraconazole (Figure 1) was used as an internal standard (IS) in both methods. The assays will be validated according to the latest ICH guidelines [20].

## 2. Materials and Methods

### 2.1. Materials and Reagents

Posaconazole was purchased from Selleckchem (Houston, TX, USA). Itraconazole was a kind gift from Nifty Labs PVT Ltd., Hyderabad, India. HPLC grade methanol and acetonitrile (Fisher Scientific UK Ltd., Loughborough, Leicestershire, UK), analytical grade potassium dihydrogen orthophosphate (Riedel-de-Haën, Germany), and high purity distilled water were used. Noxafil 40 mg/mL oral suspension (Patheon Inc., Ontario, Canada, BN 3005A) was purchased from Schering-Plough S.A.

### 2.2. Chromatographic Conditions

For the HPLC-DAD assay, the chromatography system consisted of Agilent 1200 series (quaternary pump, vacuum degasser, diode array, and multiple wavelength detector G1315 C/D and G1365 C/D) connected to a computer loaded with Agilent ChemStation Software (Agilent Technologies, Santa Clara, CA, USA). The DAD wavelength was set at 262 nm. The chromatographic separation of PSZ and itraconazole (IS) was accomplished using a Zorbax SB-C18 (4.6 × 250 mm, 5 *μ*m) column (Agilent Technologies, Santa Clara, CA, USA). The gradient elution composed of the mobile phase acetonitrile: 15 mM potassium dihydrogen orthophosphate (30 : 70 to 80 : 20, linear over 7 minutes) pumped at 1.5 mL/min. Injection volume was between 20 and 50 *μ*L. All determinations were performed at 25°C.

For the UHPLC assay, the chromatography system consisted of Agilent 1290 Infinity Binary Pump LC combined with a UV detector (Agilent Technologies, Santa Clara, CA, USA). The UV detector was set at 262 nm. The chromatographic separation of PSZ and itraconazole (IS) was accomplished using a Kinetex-C18 (2.1 × 50 mm, 1.3 *μ*m) column (Phenomenex, Torrance, CA, USA). The column temperature was set at 40 ± 0.5°C. Chromatographic data were collected and compiled by use of ChemStation software (Agilent Technologies, Santa Clara, CA, USA). The mobile phase composed of acetonitrile: 15 mM potassium dihydrogen orthophosphate (45 : 55) pumped isocratically at 0.4 mL/min. Injection volume was 5 *μ*L.

### 2.3. Standard and Stock Solutions

A 100 *μ*g/mL stock solution of PSZ was prepared by dissolving 10 mg of PSZ in 100 mL methanol. A 100 *μ*g/mL stock solution of IS was prepared by dissolving 10 mg of itraconazole in 100 mL methanol. To prepare samples for the calibration curve and validation assessment, three working solutions of 0.1, 1, and 10 *μ*g/mL of PSZ were prepared by successive 1/10 dilutions of the stock solution with methanol. All stock solutions were stored at −20°C.

### 2.4. Calibration Curve Procedures

Calibration curves were constructed using samples of PSZ and IS. The curve ranged from 5 to 50 *μ*g/mL of PSZ. IS (10 *μ*g/mL) was added to PSZ sample in a 2.5 mL polypropylene microcentrifuge tube. The volume was completed to 1 mL using methanol. The tubes were briefly vortex mixed (10 s) at high speed.

### 2.5. Oral Suspensions Extraction Procedures

A volume of 0.1 mL of the oral suspension (40 mg/mL) was diluted to 10 mL methanol (S1). 10 *μ*g/mL IS was added to 0.1 mL of S1 supernatant and diluted with methanol to a final volume of 1 mL (S2) in a 2.5 mL polypropylene microcentrifuge tube. Four replicates of S2 were freshly prepared on the day of the experiment of which 20 and 5 *μ*L were injected and chromatographed according to the previously described HPLC and UHPLC methods, respectively.

### 2.6. Validation

Validation was performed according to the latest ICH guidelines [20]. Intraday accuracy and precision of the assay were determined using three sample replicates of 5, 20, and 50 *μ*g/mL of PSZ. To permit the assessment of interday accuracy and precision, the assay was repeated on three separate days. For each daily run, concentrations were determined by comparison with a calibration curve prepared on the day of the analysis. Precision was determined using percentage coefficient of variation (CV%) and bias was assessed using mean intra- or interday percentage error of the mean. Limits of detection and quantitation were determined, at the signal to noise ratio of 3 : 1 and 10 : 1, respectively.

## 3. Results

During the development of the gradient liquid chromatographic method coupled with diode array detection for the routine quality control analysis of PSZ in its suspension dosage form, several experiments were carried out in order to optimize the choice of both the stationary and mobile phases. For optimization of the stationary phase, several reversed phase columns Zorbax SB-C8 (4.6 × 250 mm), Zorbax SB-C18 (4.6 × 250 mm), Zorbax Eclipse XDB-C18 (4.6 × 150 mm), and Brownlee Spheri-5 C18 (4.6 × 220 mm, 5 *μ*m) were tested. The best clear separation between the eluting peaks, sharper symmetric PSZ peak, and relatively shorter retention times was attained by using the Zorbax SB-C18 column; hence, it became the column of choice for this study.

Several mobile phases were also evaluated using various proportions of different aqueous phases and organic modifiers. The best mobile phase combination was acetonitrile and 15 mM potassium dihydrogen phosphate solution. Methanol was tried as an organic modifier and phosphate solution was substituted by other aqueous phases such as phosphoric acid solution. In these trials, chromatograms showed poor peak shape and/or inadequate separation between the eluting peaks. Isocratic elution of different proportions of acetonitrile and 15 mM potassium dihydrogen phosphate did not provide satisfactory separation between PSZ and IS peaks; in addition broad peaks were frequently observed. To overcome these complications, gradient elution was applied. Several gradient programs were tried and the best compromise between adequate resolution and reasonable retention times was achieved using a gradient system starting with 30% (by volume) acetonitrile ramped up linearly to 80% in 7 min and then kept at this percentage till the end of the run.

Flow rate was kept constant at 1.5 mL/min all over the run, and temperature was adjusted at 25°C. It is noteworthy to mention that the applied gradient program produced stable baseline without any drift or deformation. Quantification was achieved using diode array detection based on peak area measurement. Both PSZ and IS (itraconazole) show considerable UV absorption with prominent maximum at 262 nm which was found suitable to record all chromatograms in this study.

During the optimization of the UHPLC method, efficient separation with nondrifting baseline was achieved using a Kinetex-C18 (2.1 × 50 mm, 1.3 *μ*m) column. Again, the UV detector was set at 262 nm and a combination of acetonitrile and 15 mM potassium dihydrogen orthophosphate (45 : 55, by volume) was used as a mobile phase; however an isocratic elution was found optimum. Flow rate was adjusted at 0.4 mL/min, and temperature of the column was kept at 40°C.

**Figure 2:**
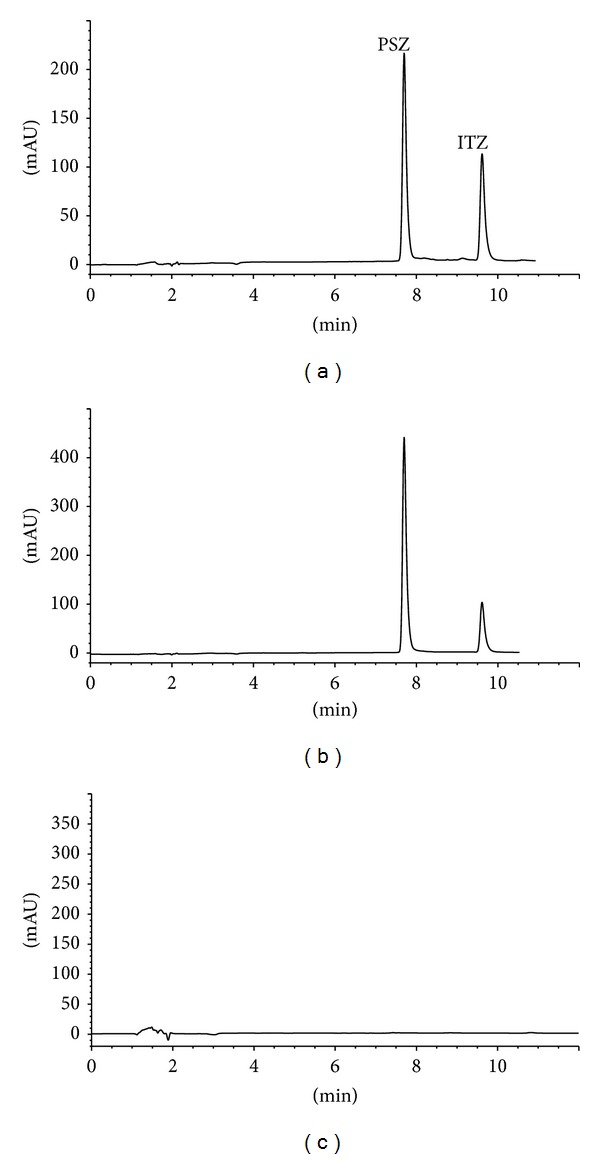
HPLC-DAD chromatograms of (a) standard posaconazole 30 *μ*g/mL and internal standard, (b) posaconazole and internal standard in suspension, and (c) blank. PSZ: posaconazole and ITZ: itraconazole.

**Figure 3:**
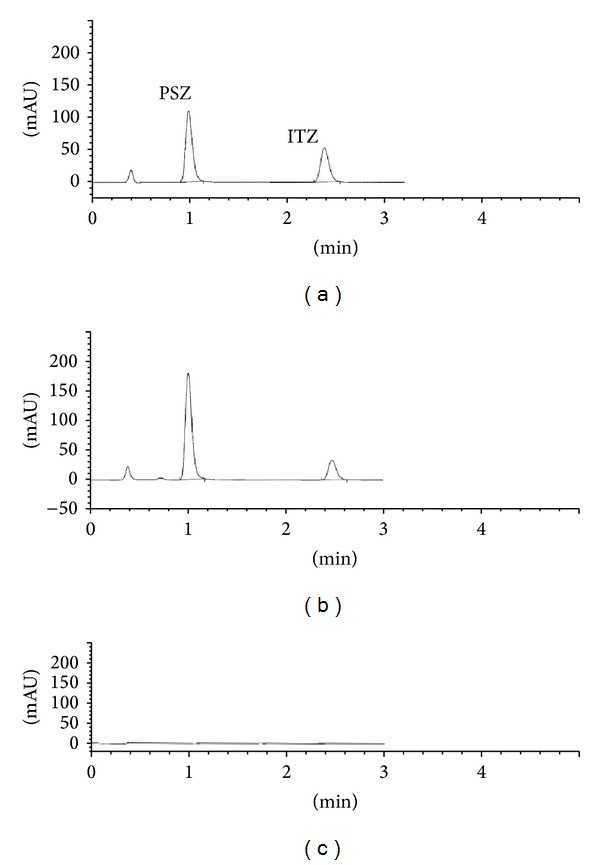
UHPLC-UV chromatograms of (a) standard posaconazole 30 *μ*g/mL and internal standard, (b) posaconazole and internal standard in suspension, and (c) blank. PSZ: posaconazole and ITZ: itraconazole.

In the HPLC-DAD assay, PSZ and the IS (itraconazole) eluted at approximately 7.7 and 9.6 min, respectively with a total analytical run time of 11 minutes (Figure 2), while in the UHPLC-UV assay, PSZ and the IS eluted at about 1.0 and 2.4 min, respectively with a total analytical run time of 3 minutes (Figure 3). The column capacity factors (*K*′) for PSZ and IS were calculated to be 3.88 and 5.13 in HPLC-DAD and 4.19 and 11.4 in UHPLC-UV, respectively. The column separation factor (*α*) was 1.25 and 2.66 for the HPLC-DAD and UHPLC-UV assays, respectively.

**Figure 4:**
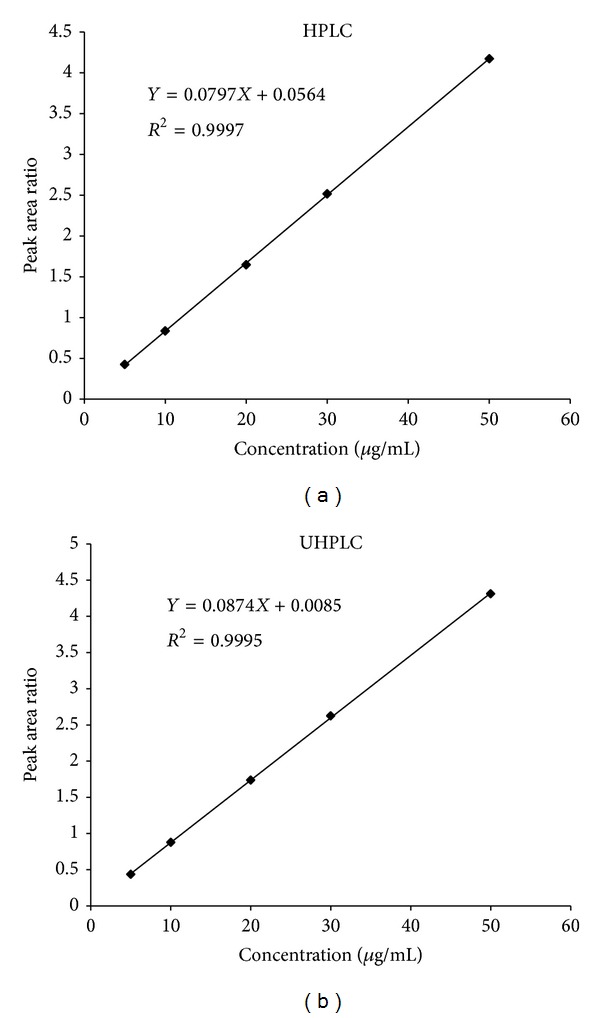
Linearity and regression for the determination of PSZ using the proposed HPLC-DAD and UHPLC-UV methods.

The proposed HPLC and UHPLC methods were validated according to the International Conference on Harmonization (ICH) guidelines on validation of analytical procedures [20]. Both assays showed highly linear relationships between the analyte/IS peak height or area ratios and concentrations ranging from 5 to 50 *μ*g/mL. The mean *r* ^2^ for the standard curves were 0.9997 and 0.9995 for the HPLC-DAD and UHPLC-UV assays, respectively. A representative standard curve using peak area ratios yielded slopes of 0.0797 and 0.0874 for HPLC-DAD and UHPLC-UV assays, respectively. The corresponding intercepts were 0.0564 and 0.0085, respectively (Figure 4).

The validation data showed the assay to be sensitive, accurate, and precise, with the intraday and interday assessment CV% less than 2% and 3% for HPLC-DAD and UHPLC-UV assays, respectively (Table 1). The mean interday error was less than 2% and 3% for HPLC-DAD and UHPLC-UV assays, respectively. The calculated limit of detection and limit of quantitation were 0.82 and 2.73 *μ*g/mL for HPLC-DAD and 1.04 and 3.16 *μ*g/mL for UHPLC-UV, respectively.

The optimized HPLC-DAD procedure was applied for the assay of PSZ in its bulk powder and its pharmaceutical formulation (Noxafil suspension). The active ingredient was extracted with the same solvent used for the preparation of the standard stock solutions (HPLC grade methanol) and then dilution was made to reach concentration levels within the specified calibration curve range. The active ingredient eluted at its specific retention time and no interfering peaks were observed from any of the inactive ingredients, as polysorbate 80, sodium benzoate, artificial cherry flavor containing benzyl alcohol, xanthan gum, and so forth (Figures 2(b) and 3(b)). The diode array detection enabled peak purity verification where no signs of coelution from any of the above excipients were detected. Recoveries were calculated using simultaneously prepared external standard. The assay results revealed satisfactory accuracy and precision as indicated from % recovery, SD, and RSD% values (Table 2). Recovery data obtained from both methods were statistically compared together using the Student's *t*- and the variance ratio *F*-tests. In both tests, the calculated values did not exceed the theoretical ones at the 95% confidence level (Table 2).

## 4. Discussion

Two novel, simple, and selective methods have been developed for the determination of PSZ in its bulk powder and suspension dosage form. The adequate recovered concentrations in addition to the low values of coefficient of variation (CV%) and percentage relative error (*E*_*r*_%) gathered in Tables 1 and 2 confirm the precision and accuracy of the developed methods for the assay of PSZ in its pure and suspension forms. In addition, the obtained *t* and *F* values indicate that there are no significant differences between the recoveries obtained from both methods. To the best of our knowledge these are the first two methods described for the determination of the drug in its dosage form. The patency and the expensive price of the drug powder and dosage form might be a reason behind that.

Our UHPLC-UV method showed similar precision and accuracy compared to the HPLC-DAD method (Tables 1 and 2); however, as previously discussed, it showed >3 fold reductions in analytical time, turnaround time, and ≥4 fold reduction in sample volume and solvents consumption with superior chromatographic separation as shown in the chromatographic parameters above [19]. Despite such great advantages, the capital investment in such new apparatuses and the availability of the HPLC in a lot of the research labs make our simple relatively quick HPLC assay an acceptable alternative as well.

Previous UHPLC methods for determination of PSZ in biological samples showed elution times ranging from 1.09 to 4.04 minutes [13, 14], whereas previous HPLC methods reported elution times ranging from 9 to 19 minutes [19, 21–24]. The only HPLC-DAD method for the determination of PSZ in bulk powder reported elution time of 8.36 minutes [15]. As such our two methods show comparable retention/run times that fall on the lower range border.

The only reported method for the determination of PSZ in bulk powder was an HPLC-DAD method using a C8 column and isocratically pumped mobile phase made of methanol : water (75 : 25), used only external standard quantitation method, had longer PSZ elution time and showed equivalent accuracy and precision [15].

Unlike fluconazole and voriconazole, PSZ is not found in a powder for reconstitution form. This makes its extraction from the suspension more challenging. The extraction of the drug occurred with no interferences from the inactive ingredients by simple dilution. Among the inactive ingredients that have UV absorption properties are the sodium benzoate and polysorbate 80. However, they were tested and proven undetected at such concentrations using our current assays. This was further confirmed using the diode-array detector whose peak purity verification of the analyte showed no signs of coelution from any of the inactive components.

## 5. Conclusion

The paper presents the first two analytical assays to determine posaconazole in its pharmaceutical dosage form. It also compares the conventional easy HPLC assay with the novel UHPLC assay. Both assays were found to be accurate, reproducible, selective, and easily applied; therefore they can be applied for the routine analysis of posaconazole in its suspension dosage form. The UHPLC-UV assay exhibited some economic and chromatographic separation superiority.

## Figures and Tables

**Table 1:** Precision and accuracy for the determination of PSZ in bulk form using the proposed HPLC-DAD and UHPLC-UV methods.

Nominal PSZ concentration *μ*g/mL	Apparatus used	Intraday mean ± SD (intraday CV%)			Interday, mean ± SD, *μ*g/mL	Interday CV%	Interday mean error%
5	HPLC	5.12 ± 0.08 (1.56)	5.02 ± 0.05 (0.99)	5.08 ± 0.10 (1.97)	5.07 ± 0.05	0.99	1.43
	UHPLC	4.99 ± 0.48 (0.96)	4.97 ± 0.14 (2.78)	5.16 ± 0.08 (1.51)	4.94 ± 0.15	1.17	0.08

20	HPLC	20.2 ± 0.1 (0.64)	19.6 ± 0.21 (1.07)	20.1 ± 0.37 (1.86)	19.9 ± 0.31	1.56	−0.14
	UHPLC	19.58 ± 0.13 (1.67)	20.31 ± 0.35 (1.74)	19.42 ± 0.26 (1.35)	19.77 ± 0.38	2.4	−1.15

50	HPLC	50.4 ± 0.45 (0.89)	49.3 ± 0.67 (1.36)	49.9 ± 0.56 (1.13)	49.9 ± 0.57	1.16	−0.22
	UHPLC	49.8 ± 0.97 (1.96)	50.5 ± 0.85 (1.69)	49.9 ± 0.66 (1.33)	50.2 ± 0.37	0.72	0.40

**Table 2:** Analysis of posaconazole in its pharmaceutical preparation (Noxafil 40 mg/mL suspension) using the proposed HPLC-DAD and UHPLC-UV methods.

Parameter	HPLC-DAD	UHPLC-UV
% Recovery ± SD^a^	101.58 ± 1.87	103.03 ± 1.50
CV%	1.84	1.46
*t*	1.21	
*F*	1.56	

^a^Mean ± standard deviation for four determinations.

Theoretical values for *t* and *F* at *P* = 0.05 are 2.45 and 9.28, respectively.
